# The DLEU2–miR-15a–16-1 Cluster Is a Determinant of Bone Microarchitecture and Strength in Postmenopausal Women and Mice

**DOI:** 10.3390/ijms252312724

**Published:** 2024-11-27

**Authors:** Sjur Reppe, Janne Elin Reseland, Vid Prijatelj, Michael Prediger, Liebert Parreiras Nogueira, Tor Paaske Utheim, Fernando Rivadeneira, Kaare M. Gautvik, Harish Kumar Datta

**Affiliations:** 1 Department of Medical Biochemistry, Oslo University Hospital, 0450 Oslo, Norway; 2 Unger-Vetlesen Institute, Lovisenberg Diaconal Hospital, 0456 Oslo, Norway; kmgautvik@gmail.com; 3 Department of Plastic and Reconstructive Surgery, Oslo University Hospital, 0424 Oslo, Norway; 4 Department of Biomaterials, Faculty of Dentistry, University of Oslo, 0317 Oslo, Norway; j.e.reseland@odont.uio.no; 5 Department of Internal Medicine, Erasmus MC, University Medical Center Rotterdam, 3015 Rotterdam, The Netherlands; 6 Blood Sciences, The Newcastle upon Tyne Hospitals NHS Foundation Trust, Royal Victoria Infirmary, Newcastle upon Tyne NE2 4HH, UK; mprediger@yahoo.com; 7 Oral Research Laboratory, Faculty of Dentistry, University of Oslo, 0317 Oslo, Norway; 8 Blood Sciences (Pathology), James Cook University Hospital, Middlesbrough TS4 3BW, UK

**Keywords:** osteoporosis, bone, *DLEU2* gene, miRNAs, eQTL, transgenic mice

## Abstract

This study explores how select microRNAs (miRNAs) influence bone structure in humans and in transgenic mice. In trabecular bone biopsies from 84 postmenopausal women (healthy, osteopenic, and osteoporotic), we demonstrate that *DLEU2* (deleted in lymphocytic leukemia 2)-encoded *miR-15a-5p* is strongly positively associated with bone mineral density (BMD) at different skeletal sites. In bone transcriptome analyses, *miR-15a-5p* levels correlated positively with the osteocyte characteristic transcripts *SOST* (encoding sclerostin) and *MEPE* (Matrix Extracellular Phosphoglycoprotein), while the related *miR-15b-5p* showed a negative association with BMD and osteoblast markers. The data imply that these miRNAs have opposite roles in bone remodeling with distinct actions on bone cells. Expression quantitative trait loci (eQTL) variants confirmed earlier *DLEU2* associations. Furthermore, a novel variant (rs12585295) showed high localization with transcriptionally active chromatin states in osteoblast primary cell cultures. The supposition that *DLEU2*-encoded miRNAs have an important regulatory role in bone remodeling was further confirmed in a transgenic mice model showing that *miR-15a/16-1*-deleted mice had significantly higher percentage bone volume and trabecular number than the wild type, possibly due to prenatal actions. However, the three-point mechanical break force test of mice femurs showed a positive correlation between strength and *miR-15a-5p/miR-16-5p* levels, indicating differential effects on cortical and trabecular bone. Moreover, these miRNAs appear to have distinct and complex actions in mice prenatally and in adult humans, impacting BMD and microstructure by regulating bone cell transcription. However, detailed interactions between these miRNAs and their downstream mechanisms in health and disease need further clarification.

## 1. Introduction

Osteoporosis is the commonest chronic bone disease, which due to a rapidly aging population is becoming a global epidemic. It is estimated that one in three women over age 50 will experience osteoporosis fractures, as will one in five men aged over 50, and worldwide, osteoporosis causes more than 8.9 million fractures annually [1]. The increased risk of osteoporotic fragility fractures is the result of loss of bone mineral density and microarchitecture deterioration. The osteoporotic bone loss occurs normally as finely coordinated resorption of bone by osteoclasts and formation of bone by osteoblasts is disrupted.

Bone metabolism is an intricate process involving a variety of cells and the respective activities of these cells are regulated by various inter- and intracellular signaling pathways and even minor aberrations in the signaling processes lead to metabolic bone diseases, including osteoporosis. Among the critical post-transcriptional modulators in bone remodeling are microRNAs (miRNAs), recognized as important players in regulation of osteoblast and osteoclast functions. The miRNAs, a form of non-coding RNA with a length of 19–25 nucleotides, are important in a wide range of processes, including cell proliferation, apoptosis, and development, thereby also impacting bone metabolic regulation. miRNAs are recognized as having fundamental regulatory functions in gene expression and cell function. Whilst the number of human miRNA candidates has been continuously increasing, only a few of them have been characterized and validated [2]. The repository miRBase holds information on over 1900 human precursors and 2656 mature miRNAs [3]. Multiple molecular mechanisms and factors are involved in miRNA regulation, including transcription, DNA methylation, miRNA processing, and biogenesis [4]. miRNAs primarily act by interacting with the 3′ untranslated region (3′ UTR) of target mRNAs, but less commonly, interaction of miRNAs with the 5′ UTR, coding sequence, and gene promoters also occurs. After binding, miRNA often induce mRNA degradation and translational repression, and the processes are influenced by the abundance of miRNA, its affinity for the target gene, and the subcellular location of miRNAs.

Over the last two decades, several in vitro and ex vivo studies have analyzed the role of miRNAs in the regulation of osteoblasts and osteoclasts and in the etiology of osteoporosis and fragility fractures [5,6,7,8,9,10,11]. These studies have identified miRNAs in the regulation of bone marrow mesenchymal stem cells (BMSCs), acting via different mechanisms and pathways targeting differentiation of bone marrow cell lineages [12]. It has been reported that overexpression of miR-15a-5p promotes apoptosis of BMSCs and reduces cell proliferation by inhibiting the Wnt/β-catenin/peroxisome proliferator-activated receptor γ (PPARγ) signaling pathway [13]. *miR-15a* has been found to target the Wnt pathway agonist *WNT3A* (Wnt Family Member 3A) and the *BCL2* (BCL2 Apoptosis Regulator) expressed in osteoblasts [14]. We reported early that *DLEU2* (deleted in lymphocytic leukemia 2), which encodes *miR-15a* and *miR16-1*, is strongly associated with BMD [15]. Next, we went on to show in weight-bearing and non-weight-bearing human postmenopausal bone that distinct subsets of noncoding RNAs are strongly associated with BMD and fracture and could explain 14% of BMD variation [16]. Here, we present the results of a study aiming to examine the role of these *DLEU2*-encoded miRNAs in human bone biopsies and in bone cell-specific knockout mice. We complemented these studies by identifying causal variants by cis-expressed quantitative trait loci (eQTL) analysis in humans.

## 2. Results

### 2.1. Correlation of miRNAs with Bone Cell-Related Transcriptomes

The expression of genes characteristic of osteoblasts, osteocytes, and osteoclasts was correlated with levels of *miR-15a-5p*, miR-16-5p and *miR-15b-5p*, respectively, in iliac trabecular bone in postmenopausal women (Table 1). *miR-15b-5p* was included in this analysis because it shares the same seed sequence as *miR-15a-5p* and *miR-16-1-5p*, and thus is likely to have similar targets [17]. Of the three miRNAs, *hsa-miR-15b-5p* was found to have more widespread significant negative correlations with almost all genes tested, except two (*COL1A1* (Collagen Type I Alpha 1 Chain) and *PDPN* (Podoplanin)) that are normally associated with cells of osteoblast lineage. On the other hand, *hsa-miR-15a-5p* showed significant positive correlations with only two genes (*SOST* and *MEPE*) characteristically expressed by osteocytes. However, there was no significant association of *hsa-miR-16-5p* with any of the other relevant genes.

### 2.2. Correlation of miRNAs with Bone Mineral Density

The relevant miRNAs *miR-15a-5p*, *miR-16-5p*, and *miR-15b-5p* were correlated with areal bone mineral density (BMD) at different skeletal sites. Interestingly, *miR-16-5p*, which did not correlate significantly with bone cell-related transcripts, also lacked significant associations with BMD at any skeletal site measured (Table 2). On the other hand, both *miR-15a-5p* and *miR-15b-5p* were significantly associated with hip, spinal, total, and head BMD. Somewhat surprisingly, whilst *miR-15a-5p* showed a positive correlation, hsa-*miR-15b-5p* and *DLEU2* were equally strongly negatively associated with BMD at all sites. The strength of correlation of *hsa-miR-15a-5p* was similar for all sites to that observed for *SOST* and significant for age-adjusted hip BMD (Z-score), as well as for BMI-adjusted Z-score. Conversely, *hsa-miR-15b-5p* showed negative correlations with *SOST* gene expression and BMD. The miRNAs *195-5p* and *497-5p*, sharing seed sequence with the other miRNAs in the table, showed a similar tendency to *hsa-miR-15a-5p* with positive correlations with BMD, although the correlations were slightly weaker.

**Table 2:** Correlations of SOST, DLEU2, and relevant miRNAs with total hip BMD T- and Z-score and BMI-adjusted Z-score in postmenopausal trans-iliac bone biopsies. The table shows Pearson correlation r values. TH: total hip; FN: femoral neck; NA: not applicable. The darker shaded cells indicate nominal *p* < 0.001; medium or lighter shaded cells indicate nominal *p* < 0.01 or *p* < 0.05, respectively. mRNA and miRNA data were obtained from 84 biopsies, while BMD data on FN, head, and total body were avail-able in 82, 77, and 78 donors, respectively. BMD data on TH and L1–L4 were available from all 84 donors. Relative miRNA levels are Ct-based (~log2 values) relative to hsa-miR-497-5p (higher number indicates higher level).

Transcript (Affymetrix ID)	Relative Levels	TH T-Score	TH Z-Score	TH BMI adj Z-Score	FN T-Score	FN Z-Score	FN BMI adj Z-Score	Head BMD (g/cm^2^)	Total Body T-Score	Total Body Z-Score	L1–L4 T-Score	L1–L4 Z-Score
*SOST* (223869_at)	NA	0.60	0.55	0.51	0.56	0.52	0.49	0.52	0.61	0.55	0.51	0.46
*DLEU2* (1556820_a_at)	NA	−0.48	−0.41	−0.39	−0.48	−0.42	−0.41	−0.43	−0.46	−0.39	−0.47	−0.41
hsa-miR-15a-5p	4.1	0.57	0.52	0.51	0.55	0.51	0.50	0.43	0.54	0.52	0.49	0.44
*hsa-miR-16-5p*	10.9	0.10	0.06	0.06	0.12	0.07	0.08	−0.07	0.06	0.01	0.08	0.04
*hsa-miR-15b-5p*	4.0	−0.46	−0.41	−0.39	−0.43	−0.39	−0.36	−0.39	−0.45	−0.41	−0.46	−0.42
*hsa-miR-195-5p*	6.5	0.28	0.23	0.18	0.26	0.20	0.17	0.18	0.28	0.23	0.29	0.23
*hsa-miR-497-5p*	0.0	0.18	0.25	0.27	0.14	0.22	0.24	0.19	0.19	0.28	0.10	0.16

**Figure 1:**
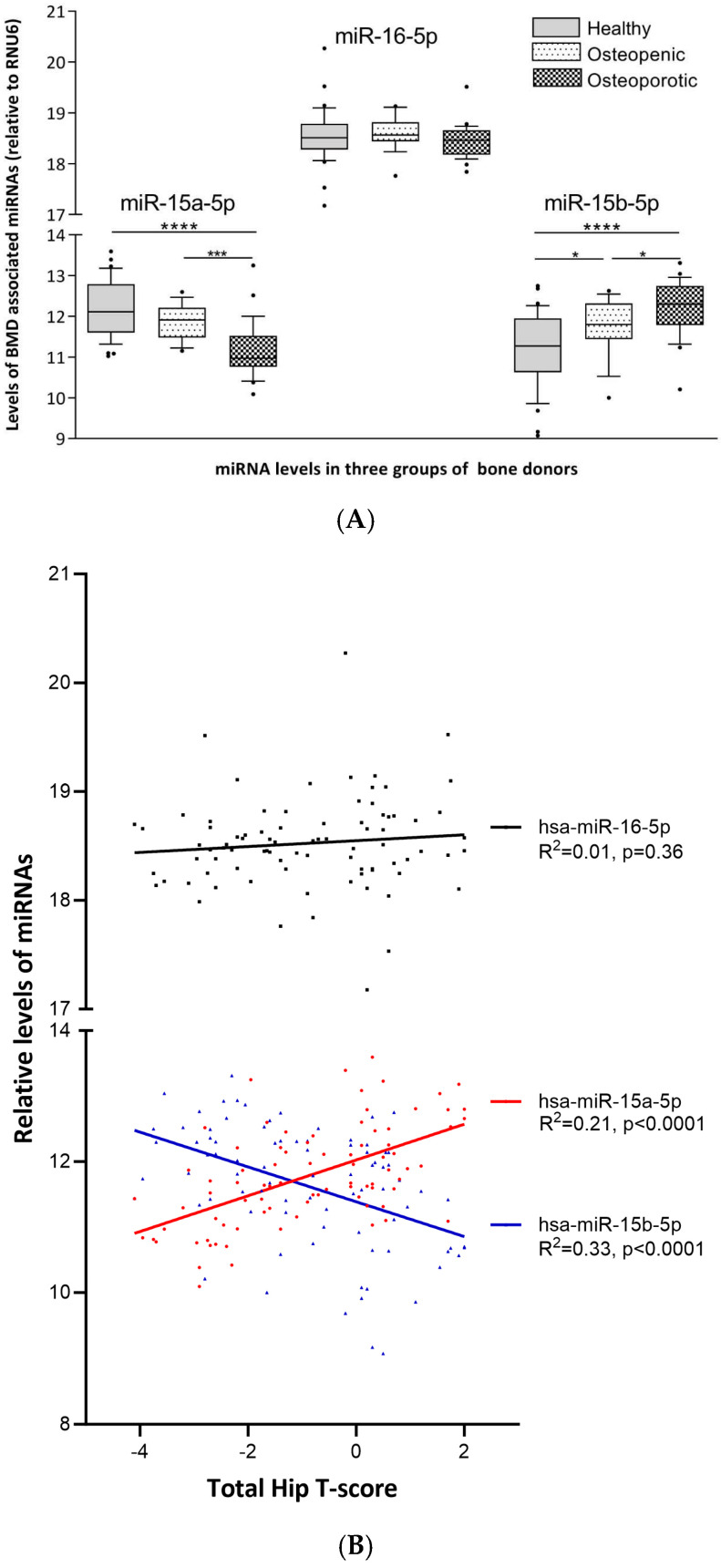
(**A**). Levels of presented miRNAs were analyzed in trans-iliac bone biopsies from healthy (n = 39), osteopenic (n = 18), and osteoporotic (n = 27) postmenopausal women, as defined by total hip BMD by a PCR-based method using TaqMan LDA arrays A and B (Applied Biosystems). (**B**)**.** Correlations between total hip bone mineral density T-scores and the relative values of miRNAs in trans-iliac bone biopsies from healthy (n = 39), osteopenic (n = 18), and osteoporotic (n = 27) postmenopausal women. *: *p* < 0.05; ***: *p* < 0.001; ****: *p* < 0.0001.

Levels of the relevant miRNAs in healthy, osteoporotic, or osteopenic women are presented in Figure 1, and reflect results from correlation studies (Table 2) with respect to levels at low/high BMD. Also, this figure demonstrates that levels of *miR-15a-5p* are similar to *miR-15b-5p*, while levels of *miR-16-5p* are several-fold higher.

### 2.3. Genotyping Results

Genotyping identified 725,497 variants in the dataset. The zCall algorithm showed an optimal z-value of 5, where 99.70% global concordance was achieved. A total of 681,594 variants were additionally recalled. Two samples were removed after a 99% call rate sample filter. An additional three samples were removed after duplicate detection. In the end, 91 samples and 642,029 variants were phased and imputed.

### 2.4. eQTL Results

**Figure 2:**
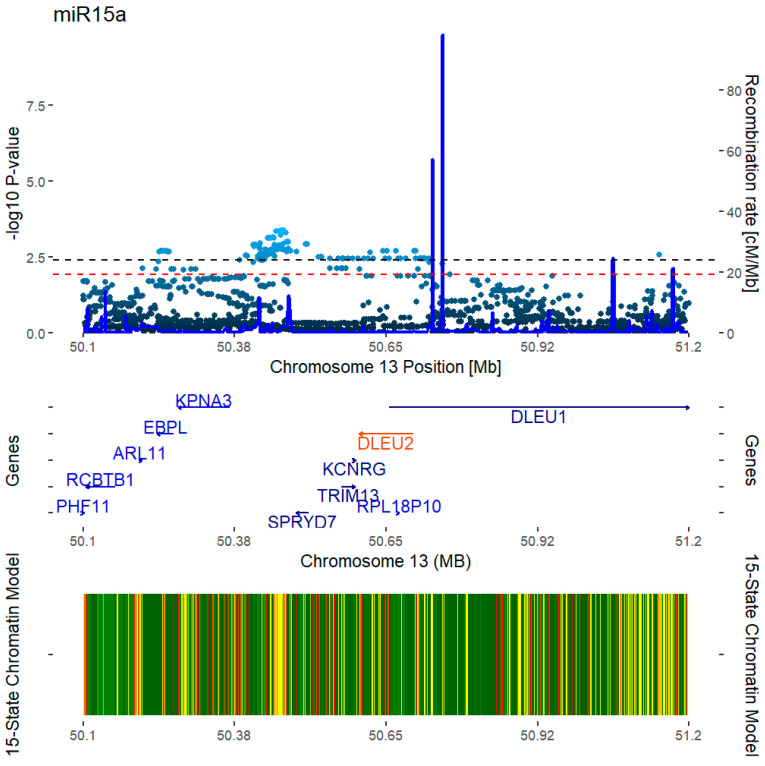
Results of the *hsa-miR15a-5p* eQTL analysis. In the top part, the association results are shown (y-axis the −log^10^ of the respective *p*-values, x-axis the genomic position on chromosome 13). Deep-blue lines show recombination rates (cM/Mb). Black and red dashed lines represent the FDR thresholds for 0.05 and 0.1, respectively. In the middle part, genes and their respective positions are shown for the cis region surrounding *DLEU2*. In the bottom part, the E129 cell line 15-state chromatin model, as provided by ROADMAP [18], is overlaid.

In total, 2039 variants were included in the analyses. In the *miR-15a-5p* analysis, 157 SNPs had a statistically significant association (Figure 2), but no SNPs reached significance (FDR < 0.05) when correlated with *miR-16-5p*. When examining the *miR-15a-5p*-associated SNPs, three distinct loci were discovered, with lead hits rs12585295, rs9526636, and rs9568300 with MAF, effect sizes, and *p*-values, as indicated in Table 3. The three SNPs were in low linkage disequilibrium (R^2^ < 0.3). Upon interrogating the phenome wide association studies (PheWASs), rs12585295 was not significantly associated with any traits, yet it had the strongest association with estimated bone mineral density (eBMD, Table 3). rs9526636 did not show significant associations with any of the traits. In contrast, rs9568300 was significantly associated with eBMD (*p*-value 2.63 × 10^−8^) and height (*p*-value 5.00 × 10^−30^).

### 2.5. Presentation of Results from Mice with Osteoblast/Osteocyte Specific Deletion of mir-15a/16

**Figure 3:**
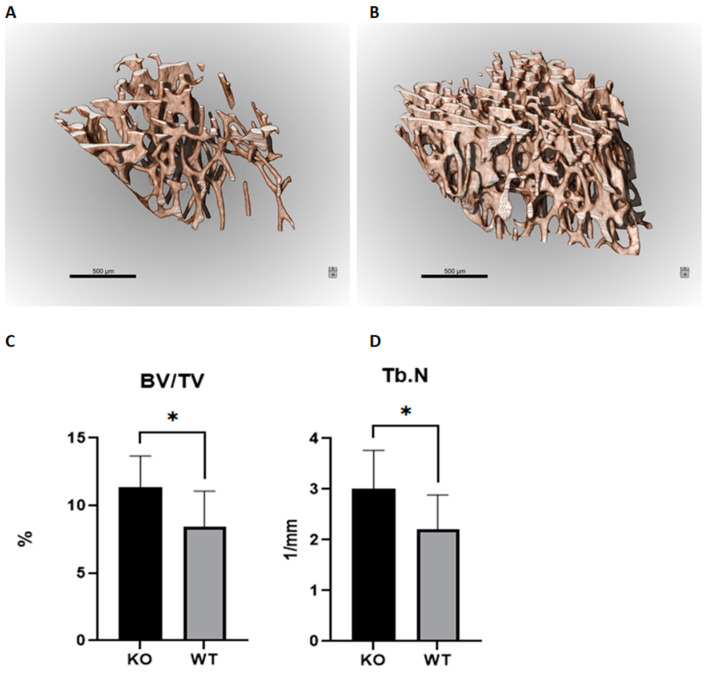
(**A**,**B**) µCT images of trabecular tibia in WT (**left**) and knockout (KO) mice (**right**). (**C**,**D**) Percentage bone volume (BV) over total volume (TV) and trabecular numbers (Tb.N), respectively. Data were obtained from 4 knockout and 19 three-month-old mice. *: *p* < 0.05.

We compared trabecular and cortical bone morphology in wild and *mir-15a/16-1^−/−^* null mice using high-resolution micro–computed tomography (µCT) imaging (Figure 3 and Table 4). Representative µCT images of trabecular bone *mir-15a/16-1^−/−^* transgenic knockout and wild-type mice are shown in Figure 3. The bone volume fraction (BV/TV) was almost 35% higher in the knockout mice, and the increased bone volume was mirrored by knockout mice having 36.7% more trabeculae than wild-type mice (Figure 3). Other parameters, such as trabecular thickness and trabecular separation, showed no significant difference.

Since the number of knockout mice was small, a firm conclusion is difficult to draw. We also present anthropometric data including BMD as a continuous variable from DXA measurements (Table 4). Both of the tested bone miRNAs were negatively associated with BMD, although miR-15a-5p did not reach significance.

### 2.6. Three-Point Break Force Test

Mouse femoral bones were subjected to a three-point fracture test as described in Methods. Due to few knockouts being available, results are instead presented with break force as a continuous variable (Table 5). Both *miR-15a-5p* and *miR-16-5p* were positively associated with break force.

## 3. Discussion

In our earliest transcriptome studies of trans-iliac bone biopsies donated by postmenopausal women with highly variable skeletal status, varying from healthy to osteoporotic with fracture, we found that *DLEU2* was present among the top eight genes showing the most significant association with BMD [15,19]. The present study explored the downstream biology of the specifically encoded *DLEU2* microRNAs *miR-15a-5p* and *miR-16-1* in addition to the structural skeletal effects observed in *mir-15a/16-1^−/−^* transgenic mice. In osteoblast/osteocyte-null mice, we observed higher bone volume and increased number of trabeculae. However, mechanical strength, which reflects mainly cortical strength, was inversely correlated with the bone levels of these miRNAs. The findings may be explained by opposite effects being exerted on cortical and trabecular bone until adult age. Thus, *mir-15a/16-1* transgenic knockout mice appear not to be representative models for the action of *DLEU2*-encoded miRNAs in adult humans. Of note, *miR-16-5p* showed no association with BMD in any skeletal sites in humans (Table 2). Further, we showed that *miR-15a-5p* was positively correlated with the osteocyte characteristic transcripts *SOST* and *MEPE* in humans, while *miR-15b-5p* showed a reciprocal association, and *miR-15a-5p* correlated, also inversely, with osteoblast characteristic transcripts. Thus, in humans, the expression of *DLEU2* and the encoded *miR-15a-5p* is closely linked with activity of the most abundant bone cells, osteocytes, recognized as the master bone regulator [20]. In contrast to *miR-15a-5p*, *miR-15b-5p* was negatively correlated with bone mineral density at all sites in humans and with almost all tested genes, except two (*COL1A1* and *PDPN*) that are normally associated with cells of osteoblast lineage. These observations strongly suggest opposing molecular mode of actions for *miR15a-5p* and *miR-15b-5p* in human bone cells. Similar contradictory results have, however, been previously observed for *SOST* gene expression, which inhibits osteoblasts activities, yet shows positive correlation with spinal and hip BMD T- and Z-scores in iliac bone biopsies from postmenopausal women [15]. *SOST* encodes the protein sclerostin, which inhibits canonical Wnt/β-catenin signaling, and the association seems counterintuitive. However, the high bone mineral density, as part of normal homeostatic response, would be expected to stimulate counter-regulatory response by the osteocytes [21] and lead to increased *SOST*, as well as *miRNA-15a-5p*. In this context, *miR-15a* has been found to target the Wnt pathway agonist *WNT3A* and also the apoptosis inhibitor *BCL2*, important for osteoblast and osteocyte apoptosis. The relevance of *mir-15a* in bone biology was further reinforced by the eQTL analysis. Our results were a supplementation of those by Zeller et al. [22], who also showed that variant rs12585295 was an eQTL for *DLEU2*. Neither the variant itself nor those in high linkage disequilibrium (LD) showed any colocalization with regulatory chromatin states [18]. rs9526636 was also a replication [23], yet with little colocalization with regulatory chromatin states. However, rs1523178, a variant in high LD with rs9526636 (R^2^ = 0.85), was shown to colocalize with transcriptionally active regulatory chromatin states [18]. Colocalization was not only seen in the osteoblast primary cells but across the majority of tissues, suggesting that the locus has a constitutive function. Both variants are ancestry-specific, being rare or non-existent in populations of African and Asian ancestry [24]. Lastly, rs9568300 was the only variant showing colocalization with GWAS results of a bone phenotype trait [25]. In addition to these known variants, the association of rs9568300 with DLEU2 expression was a novel observation. The variant and those in high LD with it were shown to be an eQTL also for *EBPL* (EBP Like) [23]. Still, the correlated variants showed high colocalization with transcriptionally active chromatin states in osteoblast primary cells, giving credence to our results. In humans, *miR-15a-5p* was positively associated with parameters of bone density and *miR-16-5p* showed no associations; however, the opposite was found in mice. The fact that bone-specific *mir-15a/16-1^−/−^* transgenic mice have a higher number of calcified trabeculae (increased BV/TV) than wild-type mice implies that in mice, the molecular action of *mir-15a-5p* is also important in bone morphogenesis and remodeling. This finding was supported by densitometric analyses in which both *miR-15a-5p* and *miR-16-5p* were inversely correlated with BMD, albeit *miR-15a-5p* did not reach significance (*p* = 0.059). At first sight, this result may seem to go against the results from the three-point break force tests showing a positive association between bone levels of the two miRNAs and fracture strength. However, cortical bone strength is a more important determinant than trabecular bone in fracture resistance [26]. Therefore, the tendency to higher cortical porosity in WT animals (almost significant) may indicate that the studied miRNAs have differential effects on cortical and trabecular bone.

In order to determine the likely target gene of *miR-15a-5p/miR-16-5p*, we used MicroT [27] to search for experimentally verified targets in humans for *hsa-miR-15a-5p* and *hsa-miR-16-5p*. Several hundreds of genes are found to be predicted, as well as experimentally supported targets, including several that have known roles in osteogenesis, namely, SMADs (SMAD Family Members), *WNT3A*, *BCL2*, *WNT4* (Wnt Family Member 4), *NFATC3* (Nuclear Factor Of Activated T Cells 3), *LRP6* (LDL Receptor Related Protein 6) (predicted only), *FOXO1* (Forkhead Box O1), and *WIF1* (WNT Inhibitory Factor 1). In this regard, it is relevant to note that previous studies have revealed that *miR-15a/16-1* targeted multiple genes that are related to the cell cycle, apoptosis, and angiogenesis, such as *CCND1* (cyclin E1), *CCND3* (cyclin D3), BCL2, MCL1 (MCL1 Apoptosis Regulator, BCL2 Family Member), WNT3A, and VEGF (Vascular Endothelial Growth Factor A) [28,29]. Indeed, previous studies have identified numerous miRNAs and likely gene targets and pathways that are involved in the regulation of bone growth, remodeling, and repair [30,31]. Other studies have identified miRNAs that target osteoblasts [32,33,34], osteocytes [35], osteoclasts [36,37,38], and pathogenesis of osteoporosis [39,40].

This study has both strengths and limitations. Unexpectedly, the results from *mir-15a/16-1* bone cell-specific knockout mice were not similar to those observed in humans. We hypothesize that this may reflect independent and partly distinct transgene actions in embryonic life. The study included few transgenic mice, and thus the conclusions are tentative, although the effect sizes were high. It is of considerable interest that these miRNAs are skeletal anabolic in mice probably before birth, but their possible developmental effects in humans are not completely resolved. However, the human results, which are the strongest part of the study, show a marked association between *miR-15b-5p* and the *DLEU2*-encoded *mir-15a–miR/16-1* cluster and bone mineral density at all the skeletal sites in postmenopausal women, as well as with characteristic bone cell markers. The detailed mechanism of actions of miR-15a-5p and miR-15b-5p are unknown, but their strong correlation with the SOST gene, a central regulator of the Wnt canonical pathway, may indicate interwoven actions.

## 4. Materials and Methods

### 4.1. Collection of Human Bone Biopsies and Human Bone miRNA Quantification

Postmenopausal trans-iliac bone biopsies (n = 84) were collected as previously described [15] using a Bordier trephine [41]. The age of donors spanned from 50 to 86 years and their bone mineral density (BMD) varied from osteoporotic to healthy. The relevant inclusion and exclusion criteria of the subjects have been previously detailed [15,18] and demographics are given in Table S1. All were free of medication or secondary diseases affecting bone metabolism. RNA was isolated as previously described [15] and quality checked using an Agilent BioAnalyzer (Agilent Technologies, Santa Clara, CA, USA). The Norwegian Regional Ethics Committee (REK 2010/2539, Norway) approved the study, all volunteers gave their written informed consent, and sampling and procedures were conducted according to the Act of Biobanking in Norway.

Quantification and normalization of mature miRNAs in human iliac bone has been described previously [16]. Briefly, total RNA (45 ng) was reverse-transcribed using Megaplex TM reverse transcription (RT) primer sets A and B (Applied Biosystems, Foster City, CA, USA), and TaqMan miRNA low-density array (LDA) A and B (Applied Biosystems) were used for PCR-based quantification.

### 4.2. Quantification of mRNAs from Postmenopausal Trans-Iliac Bone Biopsies

The same donors and methods for RNA isolation were used as described above for miRNAs. Microarray analysis of total RNA on HG-U133 plus 2.0 chips (Affymetrix Santa Clara, CA, USA) was performed as previously described [15].

The relevant data have been submitted to the EMBL-EBI (European Bioinformatics Institute) ArrayExpress repository (accession number: E-MEXP-1618).

### 4.3. Genotyping and Cis-eQTL (Expression Quantitative Trait Loci) Analyses of Iliac Bone Samples

DNA was isolated from 96 samples, either blood or bone. Genotyping was performed on GSA-MD v3 genotyping arrays (Illumina, Inc., San Diego, CA, USA). Using GenomeStudio, after initial quality control for variant filtering (cluster separation, AB R and T mean values, and variant call rates), additional quality metrics were undertaken. A total of 21 samples had a sample call rate below 97.5%, of which 4 had one below 95%. Additional quality control was performed. First, a 90% call rate sample and variant filter was applied, followed by a 97.5% call rate variant filter. A zCall routine was applied to the data, where the z-value with the best global concordance was applied [42]. Afterwards, a 99% call rate variant and sample filters were applied. Exclusion of variants was then performed based on the one-sided Hardy–Weinberg equilibrium test (cutoff *p*-value of 10^−5^). KING (Kinship-based INference for Gwas) software (version 2.3.1) was used to perform kinship analysis [43]. Within each pair of duplicate samples, the one with the lesser variant call rate was excluded. The first 20 genomic principal components were then calculated after pruning (option plink –indep-pairwise 200 5 0.05). Genotypes of 91 samples were phased using ShapeIT v2 r900 [44], followed by imputation with minimac4 [45] to the Haplotype Reference Consortium 1.1 [46]. SNPs exceeding minor allele frequency (MAF) greater than 5% and imputation quality greater than 0.3 were included in the analyses.

Using combined genetic and transcriptome data of 81 samples, cis-eQTL analyses were performed within a 1 MB window of the *DLEU2* region (chr13:50,101,269–51,199,856; ensembl GRCh37.p13) for *miR-15a-5p* and *miR-16-5p*. Associations were corrected for age, body mass index, and the first 10 genomic principal components. Analyses were performed using RVtests [47]. *p*-values of associations were adjusted for multiple testing using the Benjamini–Hochberg procedure to calculate the false-discovery rate (FDR), with FDR threshold below 0.05 being significant. Phenome-wide association (PheWAS) analysis of significant eQTL hits was performed using the Musculoskeletal Knowledge Portal [48]. Correlation of significant eQTL hits was calculated using the LDLink portal [49], using the EUR population of the 1000 Genomes Project as a basis for calculation.

### 4.4. Generation and Breeding of Transgenic Mice

The generation of bone-specific *mir-15a/16-1^−/−^* transgenic mice was achieved by employing a Cre/lox scheme. To create the *loxP* strain, a targeting vector containing an *FRT* flanked *MC1-NEO* cassette was used to insert a *loxP* site at the centromeric side of the *Mir15a/Mir16-1* cluster. A second *loxP* site was inserted between the *Mir15a*–*Mir16-1* cluster and exon 4 of the *Dleu2* gene. For the *cre* strain, a transgenic vector was generated including the human bone gamma carboxyglutamate protein (*Bglap*), otherwise known as osteocalcin (*OC*) promoter driving expression of nuclear localized *cre-recombinase*. Therefore, the human bone gamma carboxyglutamate protein (Bglap) promoter/enhancer directs OC-cre transgenic mice and Cre expression. Mice hemizygous for the transgene are viable and fertile. The female mouse jax strain B6N.FVB-Tg(*BGLAP-cre*) was mated with the homozygous *loxP*-flanked strain B6.129S-Mirc30tm1.1Rdf/. This is predicted to generate approximately 50% of the offspring that are heterozygous for the *loxP* allele and hemizygous/heterozygous for the *cre* transgene. This progeny was mated with homozygous *loxP*-flanked mice.

### 4.5. Anthropometric Markers of Transgenic Mice

BMD and other anthropometric markers were measured by dual-energy X-ray absorptiometry (DXA) using a PIXImus densitometer (G.E. Medical Systems, Lunar Division, Madison, WI, USA). BMD measurements included total BMD (excluding the head and tail), L1–L6 vertebra (spinal BMD), and entire femur (femoral BMD). Body weight was measured at the time of the DXA scan. Calibration was performed using a standard control phantom before scanning, as recommended by the manufacturer.

### 4.6. Three-Point Break Force Test

Three-point break force tests were performed on femurs from three-month-old mice using a single-column Lloyd LRX testing system (Lloyd Instruments, Fareham, UK). The mechanical test was performed at a displacement rate of 0.01 mm/s and a sampling rate of 20 Hz.

### 4.7. Isolation of Mouse Bone DNA and RNA

*Bone RNA isolation:* Femurs from three-month-old mice were freed from muscle, ligaments, and bone marrow and placed in 2 mL micro-centrifuge tubes with 700 μL Qiazol (Qiagen, Beverly MA, USA) and one stainless steel bead (5 mm in diameter). The tubes were shaken on a Tissue Lyzer (Qiagen) for 2 min at 25 Hz. The homogenate was rested at 22 °C for 20 min, mixed with 140 μL chloroform, and centrifuged (12,000× *g*, 15 min). RNA was isolated from the upper aqueous phase using the miRNeasy micro kit (Qiagen) according to the manufacturer’s recommendations.

*Bone DNA isolation*: Humeri from 3-month-old mice/femurs from 1-month-old mice were freed from muscle, ligaments, and bone marrow and incubated at 56 °C for 2 h in lysis cocktail A (Bone DNA Extraction Kit (AX6780, Promega, Madison, WI, USA). DNA was purified from the resulting solution using a Monarch Genomic DNA Purification Kit (New England Biolabs, Ipswich, MA, USA) according to the manufacturer’s recommendations.

### 4.8. Genotyping of Mouse Bone DNA

**Figure 4:**
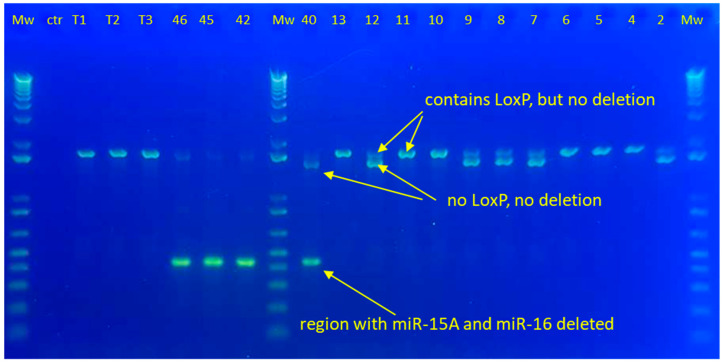
Genotyping of mouse bone DNA. The picture shows a typical 1.2% agarose gel for separation of PCR products, including molecular weight standards (Mw) and control (ctr). Text indicates genotype of the differentially sized product verified by Sanger sequencing.

To genotype DNA from femoral bone tissue of the transgenic mice, we used primers flanking the loxP sites (forward CTCGAGCCTTGGTGGTACTG; reverse GCTTTGTCCTATGGATTTGGCT). We used PCR cycling conditions favoring amplification of the shorter knocked-out region (94 °C for 25 s followed by 32 cycles of 94 °C for 25 s, 53 °C for 15 s, and 68 °C for 20 s) using OneTaq Hot Start 2X Master Mix with Standard Buffer (New England Biolabs Ipswich, MA, USA). The resulting PCR products were identified by agarose gel electrophoresis. To verify the identity of the PCR products, the differentially sized PCR products were cut out from a preparative gel and purified using the Monarch Gel Extraction Kit (New England Biolabs Ipswich, MA, USA). The purified PCR products were then subjected to Sanger sequencing with the same primers as used for PCR. Figure 4 shows a typical agarose gel with the resulting three different PCR products.

### 4.9. PCR Quantification of Mouse Bone miRNAs

cDNA from mouse bone was synthesized using Invitrogen MultiScribe™ Reverse Transcriptase (Fisher Scientific, Oslo, Norway) according to the manufacturer’s recommendations. We performed real-time PCR using TaqMan™ Fast Universal PCR Master Mix (2X) and No AmpErase UNG (Thermo Fisher Scientific, Waltham, MA, USA) with a ViiA 7 Real-Time PCR System (Thermo Fisher Scientific). TaqMan assays are presented in Table 6. U6 small nuclear RNA (RNU6) was used as endogenous standard. The cDNA was amplified using recommended PCR cycling conditions (95 °C for 10 min followed by 40 cycles of 95 °C for 15 s and 60 °C for 1 min). The relative amounts of miRNAs compared to RNU6 (dCT values) were calculated by subtracting the average Ct value for RNU6 from the average Ct value of the tested miRNA in every sample.

### 4.10. µCT

Tibial bones were wrapped in wet gauze and put inside an Eppendorf^®^ tube for micro-CT scan. They were scanned using the Skyscan 1172 microCT system from Bruker (Kontich, Belgium). A total of 902 projections were evenly acquired with a final isotropic voxel size of 5.0 µm, with camera binning 2 × 2 of a CCD camera (11 Mpixel CCD detector) (Ximea, Münster, Germany), 60 kV accelerating voltage, 168 μA current, and a 0.5 µm aluminum filter placed in front of the beam to remove most of the undesirable low-energy X-rays from the beam. During acquisition, the sample was rotated by 360° about its vertical axis at a step size of 0.40°, with an exposure time of 885 ms per projection, taking an average of 3 frames to reduce the noise. The tomograms were reconstructed with NRecon v.1.7.1.0 software (Bruker), which uses a filtered back-projection algorithm with the following parameters: ring artefact correction of 12, beam hardening correction of 35%, and no smoothing. The porosity analysis was performed using CTAnalyser (CTAn 1.20.3, Bruker).

## Figures and Tables

**Table 1:** Correlations between key bone cell type marker transcripts and miRNAs. Numbers are Pearson correlations of r-values between levels of human bone miRNAs or *DLEU2* and key bone cell-type markers. Shaded cells indicate nominally significant values (*p* < 0.05).

Affy ID	Gene Symbol	*hsa-miR-15a-5p*	*hsa-miR-16-5p*	*hsa-miR-15b-5p*	*DLEU2*
Osteoblast markers					
1556499_s_at	*COL1A1*	−0.04	0.01	−0.15	0.02
236028_at	*IBSP*	0.10	−0.04	−0.30	−0.01
200665_s_at	*SPARC*	0.01	−0.01	−0.23	0.06
206956_at	*BGLAP*	0.06	−0.03	−0.26	0.00
207173_x_at	*CDH11*	0.16	−0.16	−0.34	−0.10
205911_at	*PTH1R*	0.02	−0.10	−0.26	0.14
Osteocyte markers					
226658_at	*PDPN*	−0.11	−0.05	−0.13	−0.12
221150_at	*MEPE*	0.54	−0.07	−0.59	−0.26
223869_at	*SOST*	0.56	0.01	−0.59	−0.26
Osteoclast markers					
204638_at	*ACP5 (TRAP)*	0.00	−0.04	−0.11	0.06
202450_s_at	*CTSK*	0.06	−0.10	−0.23	−0.01
207887_s_at	*CALCR*	−0.17	0.13	0.08	0.06
1554503_a_at	*OSCAR*	−0.10	−0.02	0.09	0.07

**Table 3:** Characteristics of the identified SNPs. MAF: minimal allele frequency; eBMD: estimated bone mineral density.

SNP	MAF	Effect Size	*p*-Value	PheWAS eBMD
Rs12585295	0.40	0.38	4.28 × 10^−4^	3.63 × 10^−5^
Rs9526636	0.17	0.54	1.99 × 10^−3^	
Rs9568300	0.17	−0.49	2.07 × 10^−3^	2.63 × 10^−8^

**Table 4:** Correlations of mouse femoral bone miRNAs with BMD and anthropometric markers. The table presents Pearson correlation r and *p* values from correlations of three-month-old mice femoral miRNA levels with indicated markers. All values except weight were obtained by a PIXImus densitometer. ns: not significant, n = 23 (including KO and WT). Shaded cells indicate *p* > 0.05.

	miR-15a-5p		miR-16-5p	
	R	*p*-Value	r	*p*-Value
Weight (g)	0.39	0.066	0.21	ns
BMD total	−0.40	0.059	−0.52	0.011
BMC total	0.07	ns	0.02	ns
Lean total	0.28	ns	0.08	ns
Fat total	0.27	ns	0.12	ns
BMD femur left	−0.12	ns	−0.16	ns
BMC femur left	−0.23	ns	−0.29	ns
Lean femur left	−0.35	0.098	−0.21	ns
Fat femur left	0.19	ns	0.18	ns
BMD lumbar	−0.32	ns	−0.28	ns
BMC lumbar	−0.34	ns	−0.27	ns
Lean lumbar	−0.28	ns	−0.34	ns
Fat lumbar	0.00	ns	0.00	ns

**Table 5:** Three-point break force test. The table indicates Pearson correlation r and *p* values from correlations of three-month-old mouse femoral miRNA levels with indicated parameters. Data are without outliers (mice #26 and #29), n = 21 (including KO and WT). “mm” refers to length of femur. Shaded cells indicate *p* < 0.05.

	miR-15a-5p		miR-16-5p	
	r	*p*-Value	r	*p*-Value
Break force (N)	0.46	0.036	0.49	0.024
Elastic modulus (MPa)	0.30	0.186	0.46	0.036
Stiffness (N/mm)	0.40	0.070	0.43	0.052

**Table 6:** TaqMan assays.

Transcript	TaqMan™ MicroRNA Assay
mmu-miR-15a-5p	mmu482962_mir
mmu-miR-16-5p	mmu482960_mir
RNU6	Mm01160338_m1

## Data Availability

The data for postmenopausal biopsies have been submitted to the European Bioinformatics Institute (EMBL-EBI; ArrayExpress repository, ID: E-MEXP-1618). The data on male biopsies are accessible through accession number E-MEXP-2219. The scripts for analysis are available at https://github.com/Bioinformatics-Support-Unit/python-scripts/tree/master/zic1 (accessed on 26 April 2016).

## Supplementary Materials

The following supporting information can be downloaded at https://www.mdpi.com/article/10.3390/ijms252312724/s1.
