# Supplementary material for: The DLEU2–miR-15a–16-1 Cluster Is a Determinant of Bone Microarchitecture and Strength in Postmenopausal Women and Mice
Source: Int J Mol Sci. 2024 Nov 27;25(23):12724. doi: 10.3390/ijms252312724 (PMC11641114; doi:10.3390/ijms252312724)
Supplement: Supplementary file 1 [file ijms-25-12724-s001.zip › ijms-3294184-supplementary.pdf]

*Article*

# The DLEU2/miR-15a/16-1cluster is a determinant of bone microarchitecture and strength in postmenopausal women and mice

**Reppe S.[1,2,3], Reseland J.E.[4], Prijatelj V.[5], Prediger M[6], Nogueira L.P.[7], Utheim, T.P.[1,3], Rivadeneira F.[5], Gautvik K.M.[2], Datta H.K.[8,*]**

[1] Department of Medical Biochemistry, Oslo University Hospital, 0450 Oslo, Norway;
[2] Unger-Vetlesen Institute, Lovisenberg Diaconal Hospital, 0456 Oslo, Norway
[3] Department of Plastic and Reconstructive Surgery, Oslo University Hospital, 0424 Oslo, Norway
[4] Department of Biomaterials, Faculty of Dentistry, University of Oslo, Oslo, Norway
[5] Department of Internal Medicine, Erasmus MC, University Medical Center Rotterdam, Rotterdam, The Netherlands
[6] Blood Sciences, The Newcastle upon Tyne Hospitals NHS Foundation Trust, Royal Victoria Infirmary, Newcastle upon Tyne NE2 4HH, UK.
[7] Oral Research Laboratory, Faculty of Dentistry, University of Oslo, Oslo, Norway
[8] Blood Sciences (Pathology), James Cook University Hospital, Middlesbrough, TS4 3BW, UK.
* Correspondence: * harish.datta1@nhs.net.

**Supplementary Table S1** Characteristics of postmenopausal bone donors

| | **Osteoporotic (n=27)** | | **Osteopenic (n=18)** | | **Healthy (n=39)** | |
|---|---|---|---|---|---|---|
| | **Mean** | **SD** | **Mean** | **SD** | **Mean** | **SD** |
| **Antropometric markers** | | | | | | |
| **Age (years)** | **69.41** | **10.61** | 62.37 | 8.26 | 62.22 | 8.24 |
| **Body mass index (kg/m$^2$)** | **22.56** | **3.04** | 23.54 | 2.67 | 25.57 | 3.90 |
| **L2-L4 g/cm2** | **0.77** | **0.12** | 0.83 | 0.06 | 1.29 | 0.09 |
| **L2-L4 Z-score** | **-1.97** | **0.82** | -1.98 | 0.69 | 1.68 | 0.99 |
| **Total Hip g/cm2** | **0.68** | **0.09** | 0.85 | 0.09 | 1.06 | 0.09 |
| **Total Hip Z-score** | **-1.27** | **0.62** | -0.28 | 0.78 | 1.30 | 0.94 |
| **Total body g/cm2** | **0.93** | **0.07** | 1.01 | 0.06 | 1.20 | 0.06 |
| **Total body Z-score** | **-1.09** | **0.82** | -0.41 | 0.79 | 1.63 | 0.92 |
| **Biochemical markers** | | | | | | |
| **S-Vitamin K(µg/l)** | **0.46** | **0.23** | 0.61 | 0.44 | 0.51 | 0.24 |
| **S- intact PTH (pmol/l)** | **5.14** | **2.53** | 4.04 | 1.58 | 4.16 | 1.92 |
| **S-Ca$^{2+}$ corr. (mmol/l)** | **1.25** | **0.05** | 1.27 | 0.04 | 1.24 | 0.04 |
| **S-25(OH)D3 (nmol/l)** | **87.79** | **44.04** | 92.33 | 32.41 | 76.24 | 26.98 |
| **S-Osteocalcin (nmol/ml)** | **1.62** | **0.63** | 1.51 | 0.66 | 1.34 | 0.54 |
| **S-1-CTP (CTx) (µg/l)** | **4.67** | **2.12** | 3.79 | 0.83 | 3.64 | 0.97 |
| **S-bone specific ALP (U/l)** | **28.95** | **9.39** | 26.60 | 8.92 | 20.95 | 8.34 |
| **S-phosphate (mmol/l)** | **1.18** | **0.15** | 1.17 | 0.16 | 1.20 | 0.17 |
| **U-NTx (mmol/l)** | **67.86** | **34.05** | 67.71 | 34.86 | 51.63 | 24.57 |
| **U-DPD (mM DPD/mM Cr)** | **7.72** | **2.42** | 7.59 | 1.97 | 6.88 | 2.55 |

Initially, 301 non-related postmenopausal ethnic Norwegian women (50–86 years) were consecutively recruited, at the Lovisenberg Diaconal Hospital, the Out-patient Clinic, Oslo, but 178 were rejected due to medication or diseases. Of the 123 enrolling the study, 23 later declined to participate. All persons with underlying diseases other than osteoporosis or receiving medication (past or present) possibly affecting bone remodelling or representing secondary causes of osteoporosis (e.g. steroid medications) were excluded.

The included participants showed normal endocrine, clinical, biochemical, and nutritional status, and all had been postmenopausal for at least two years. All groups (Osteoporotic with at least one fragility fracture, Osteopenic and Healthy included previous estrogen users, but they had been without

medication for at least two years prior to this study, except one (in the Osteoporosis group) who had been without the drug for six months. This is a brief summary of information presented earlier (PMID: 19922823 and PMID: 21452281).
